# Supplementary material for: L‒asparaginase activity in some endophytic fungi: Glutaminase‒free and low urease co‒activities
Source: PLoS One. 2026 Feb 13;21(2):e0339829. doi: 10.1371/journal.pone.0339829 (PMC12904411; doi:10.1371/journal.pone.0339829)
Supplement: S1 Table — (PDF) [file pone.0339829.s001.pdf]

S1 Table. Characteristics of the studied endophytic fungal isolates (names, host sources, and accession numbers of ITS1-5.8S-ITS2 rDNA sequences).

**Isolates**	**Host**	**Accession No.**
Cytospora leucostoma	*Taxus baccata* L.	KF573982
anamorphic *Xylaria*		KF573972
Chaetomium globosum	*Pistacia vera* L.	KP641141
Fusarium chlamydosporum		KP641161
Alternaria malorum		-
Cytospora chrysosperma		KP641137
Ulocladium sp.		KP641145
Scopulariopsis brevicaulis		KP641165
Alternaria brassicae		KP641144
Nigrospora oryzae		KP641153
Acremonium sclerotigenum		KP641158
Chaetomium elatum		KP641140
Byssochlamys nivea		KP641160
Neoscytalidium dimidiatum		KP641157
Aspergillus tamarii		KP641150
Aspergillus flavus		-

Aspergillus nidulans		-
Penicillium chrysogenum		KP641164
Trichoderma longibrachiatum		KP641159
Aspergillus niger		-
Nectria sp.	*Prunus cerasus* L.	-
Acremonium egyptiacum		KY472286
Trichothecium roseum		KX761894
Fusarium fujikuroi		KY472302
Chalastospora gossypii		KX761889
Alternaria multiformis		KY472288
Coniolariella limonispora		KY472296
Cladosporium perangustum		KY472294
Alternaria tenuissima		KY472290
Acremonium sclerotigenum		KX761888
Fusarium prolifratum	*Punica granatum* L.	MF288745
Fusarium fujikuroi		MF288740

Fusarium solani		MF288736
Bipolaris sorokiniana		MF288734
Sporormiella australis		MF288744
Aureobasidium pullulans		MF288729
Chaetomium globosum		MF288735
Cladosporium herbarum		MF288739
Cladosporium cladosporioides		MF288749
Neoscytalidium novaehollandiae		MF288738
Aureobasidium melanogenum		MF288750
Alternaria atra		MF288747
Pelectosphaerella cucumerina		MF288743
Cytospora punicae		MF288730
Purpureocillium lilacinum		MF288731
Alternaria gaisen		MF288746

Trichothecium roseum		MF288732
Alternaria longipes		MF288741

"-": Lack of information.
