# Supplementary material for: L‒asparaginase activity in some endophytic fungi: Glutaminase‒free and low urease co‒activities
Source: PLoS One. 2026 Feb 13;21(2):e0339829. doi: 10.1371/journal.pone.0339829 (PMC12904411; doi:10.1371/journal.pone.0339829)
Supplement: S2 Table — According to Levene’s test, the variances between enzyme activity in the culture media with and without substrate are equal and independent two-tailed t‒test is significant in studied isolates except in Cytospora leucostoma G88 (p‒value = 0.303). (PDF) [file pone.0339829.s002.pdf]

S2 Table. Results of independent samples t-test analysis. According to Levene's test, the variances between enzyme activity in the culture media with and without substrate are equal and independent two-tailed t-test is significant in studied isolates except in *Cytospora leucostoma* G88 (p-value = 0.303).

		Levene's Test for Equality of Variances		t-test for Equality of Means						
									95% Confidence Interval of the Difference	
		F	Sig.	t	df	Sig. (2-tailed)	Mean Difference	Std. Error Difference	Lower	Upper
**Kr5-2**	Equal variances assumed	1.298	0.318	-27.172	4	5.9×10^{-8}	-10.35667	0.38115	-11.41492	-9.29842
	Equal variances not assumed			-27.172	2.526	0.000	-10.35667	0.38115	-11.70925	-9.00408
**IH1-2**	Equal variances assumed	0.162	0.708	-76.240	4	4.6×10^{-9}	-6.27667	0.08233	-6.50524	-6.04809
	Equal variances not assumed			-76.240	3.841	0.000	-6.27667	0.08233	-6.50902	-6.04432
**EL1**	Equal variances assumed	0.199	0.679	-85.842	4	1.8×10^{-9}	-3.68667	0.04295	-3.80591	-3.56743
	Equal variances not assumed			-85.842	3.963	0.000	-3.68667	0.04295	-3.80634	-3.56699

**Zn8-2**	Equal variances assumed	4.377	0.105	-4.958	4	0.008	-1.47333	0.29717	-2.29841	-0.64825
	Equal variances not assumed			-4.958	2.485	0.024	-1.47333	0.29717	-2.54024	-0.40643
**G88**	Equal variances assumed	1.652	0.268	-1.180	4	0.303	-0.30667	0.25983	-1.02807	0.41473
	Equal variances not assumed			-1.180	2.567	0.336	-0.30667	0.25983	-1.21838	0.60504
