# Supplementary material for: L‒asparaginase activity in some endophytic fungi: Glutaminase‒free and low urease co‒activities
Source: PLoS One. 2026 Feb 13;21(2):e0339829. doi: 10.1371/journal.pone.0339829 (PMC12904411; doi:10.1371/journal.pone.0339829)
Supplement: S3 Table — The sample size consisted of 12 endophytic fungal isolates and the experiment was conducted in triplicate. (PDF) [file pone.0339829.s003.pdf]

S3 Table. One-way ANOVA results indicated a significant difference ($p < 0.05$) among fungal isolates in each culture media in L-asparaginase production. The sample size consisted of 12 endophytic fungal isolates and the experiment was conducted in triplicate.

		Sum of Squares	df	Mean Square	F	Sig.
**Sucrose proline agar**	Between Groups	140.062	11	12.733	517.014	3.0×10^{-36}
	Within Groups	0.591	24	0.025		
	Total	140.653	35			
**Mineral salts agar**	Between Groups	307.906	11	27.991	249.888	3.0×10^{-30}
	Within Groups	2.688	24	0.112		
	Total	310.595	35			
**Asthana and Hawker culture medium A**	Between Groups	135.987	11	12.362	410.732	3.0×10^{-33}
	Within Groups	0.722	24	0.030		
	Total	136.709	35			
**Elliott agar**	Between Groups	215.804	11	19.619	449.186	2.0×10^{-33}
	Within Groups	1.048	24	0.044		
	Total	216.852	35			
**Brown agar**	Between Groups	260.766	11	23.706	324.925	5.0×10^{-32}
	Within Groups	1.751	24	0.073		
	Total	262.517	35			
**Dox agar**	Between Groups	116.208	11	10.564	373.749	1.0×10^{-31}
	Within Groups	0.678	24	0.028		
	Total	116.886	35			
**Cerelose ammonium nitrate**	Between Groups	14.694	11	1.336	289.562	2.0×10^{-31}
	Within Groups	0.111	24	0.005		
	Total	14.805	35			
**Citrate agar culture medium**	Between Groups	256.630	11	23.330	396.300	5.0×10^{-32}
	Within Groups	1.413	24	0.059		
	Total	258.043	35			
**Kuehner basal culture medium**	Between Groups	45.987	11	4.181	316.398	1.0×10^{32}
	Within Groups	0.317	24	0.013		

	Total	46.304	35			
**Piefer, Humphrey, and Acree culture medium**	Between Groups	27.933	11	2.539	190.973	2.0×10^{-28}
	Within Groups	0.319	24	0.013		
	Total	28.253	35			
**MCD**	Between Groups	157.544	11	14.322	323.730	5.0×10^{-32}
	Within Groups	1.062	24	0.044		
	Total	158.605	35			
