# Supplementary material for: L‒asparaginase activity in some endophytic fungi: Glutaminase‒free and low urease co‒activities
Source: PLoS One. 2026 Feb 13;21(2):e0339829. doi: 10.1371/journal.pone.0339829 (PMC12904411; doi:10.1371/journal.pone.0339829)
Supplement: S4 Table — The sample size consisted of 11 culture media (MCD plus 10 additional culture media) and the experiment was conducted in triplicate. (PDF) [file pone.0339829.s004.pdf]

S4 Table. One-way ANOVA results indicated a significant difference ($p < 0.05$) among culture media for L-asparaginase production in each isolate. The sample size consisted of 11 culture media (MCD plus 10 additional culture media) and the experiment was conducted in triplicate.

		Sum of Squares	df	Mean Square	F	Sig.
**EL1**	Between Groups	327.397	10	32.740	217.877	5.0×10^{-28}
	Within Groups	3.306	22	0.150		
	Total	330.703	32			
**Kr5-2**	Between Groups	64.341	10	6.434	60.614	2.0×10^{-14}
	Within Groups	2.335	22	0.106		
	Total	66.676	32			
**IH1-2**	Between Groups	496.328	10	49.633	1532.449	2.0×10^{-47}
	Within Groups	0.713	22	0.032		
	Total	497.040	32			
**C**	Between Groups	47.362	10	4.736	197.391	6.0×10^{-28}
	Within Groups	0.528	22	0.024		
	Total	47.890	32			
**SAA10**	Between Groups	19.203	10	1.920	655.312	2.0×10^{-38}
	Within Groups	0.064	22	0.003		
	Total	19.267	32			
**IIV3-3**	Between Groups	6.198	10	0.620	12.107	3.8×10^{-5}
	Within Groups	1.126	22	0.051		
	Total	7.324	32			
**I27**	Between Groups	41.198	10	4.120	93.304	2.5×10^{-15}
	Within Groups	0.971	22	0.044		
	Total	42.169	32			
**URA1**	Between Groups	37.252	10	3.725	531.941	2.0×10^{-36}
	Within Groups	0.154	22	0.007		
	Total	37.406	32			
**KhDS2-3**	Between Groups	9.324	10	0.932	409.146	5.0×10^{-35}
	Within Groups	0.050	22	0.002		

	Total	9.374	32			
**EES2-2**	Between Groups	87.302	10	8.730	222.865	2.0×10^{-28}
	Within Groups	0.862	22	0.039		
	Total	88.164	32			
**Zn8-2**	Between Groups	315.182	10	31.518	1351.308	5.0×10^{-47}
	Within Groups	0.513	22	0.023		
	Total	315.695	32			
**G88**	Between Groups	20.461	10	2.046	573.188	5.0×10^{-37}
	Within Groups	0.079	22	0.004		
	Total	20.540	32			
