# Supplementary material for: L‒asparaginase activity in some endophytic fungi: Glutaminase‒free and low urease co‒activities
Source: PLoS One. 2026 Feb 13;21(2):e0339829. doi: 10.1371/journal.pone.0339829 (PMC12904411; doi:10.1371/journal.pone.0339829)
Supplement: S5 Table — (PDF) [file pone.0339829.s005.pdf]

S5 Table. Dunnett's t-tests treat one group (MCD culture medium) as a control, and compare all other groups (ten different culture media) against it.

Dependent Variable	(I) culture_media	(J) culture_media	Mean Difference (I-J)	Std. Error	Sig.	95% Confidence Interval Lower Bound	Upper Bound
**EL1**	Scrose proline agar	MCD	-1.70000*	0.31651	0.000	-2.6360	-0.7640
	Mineral salts agar	MCD	0.92000	0.31651	0.056	-0.0160	1.8560
	Asthana and Hawker medium A	MCD	-3.11000*	0.31651	0.000	-4.0460	-2.1740
	Elliott's agar	MCD	0.68000	0.31651	0.239	-0.2560	1.6160
	Brown's agar	MCD	2.13000*	0.31651	0.000	1.1940	3.0660
	Dox agar	MCD	-1.02000*	0.31651	0.028	-1.9560	-0.0840
	Cerelose ammonium nitrate	MCD	-6.58000*	0.31651	0.000	-7.5160	-5.6440
	Citrate agar medium	MCD	2.97667*	0.31651	0.000	2.0407	3.9126
	Kuehner's basal medium	MCD	-4.43000*	0.31651	0.000	-5.3660	-3.4940
	Piefer, Humphrey and Acree's medium	MCD	-6.58000*	0.31651	0.000	-7.5160	-5.6440
**Kr5-2**	Scrose proline agar	MCD	-1.82667*	0.26602	0.000	-2.6133	-1.0400
	Mineral salts agar	MCD	1.79333*	0.26602	0.000	1.0067	2.5800
	Asthana and Hawker medium A	MCD	0.62000	0.26602	0.173	-0.1667	1.4067
	Elliott's agar	MCD	-1.73000*	0.26602	0.000	-2.5167	-0.9433
	Brown's agar	MCD	0.37667	0.26602	0.670	-0.4100	1.1633

	Dox agar	MCD	-0.90667*	0.26602	0.019	-1.6933	-0.1200
	Cerelose ammonium nitrate	MCD	-3.02333*	0.26602	0.000	-3.8100	-2.2367
	Citrate agar medium	MCD	-0.06667	0.26602	1.000	-0.8533	0.7200
	Kuehner's basal medium	MCD	-1.26000*	0.26602	0.001	-2.0467	-0.4733
	Piefer, Humphrey and Acree's medium	MCD	-2.55000*	0.26602	0.000	-3.3367	-1.7633
**IH1-2**	Scrose proline agar	MCD	2.22000*	0.14694	0.000	1.7855	2.6545
	Mineral salts agar	MCD	5.13000*	0.14694	0.000	4.6955	5.5645
	Asthana and Hawker medium A	MCD	0.85000*	0.14694	0.000	0.4155	1.2845
	Elliott's agar	MCD	2.94333*	0.14694	0.000	2.5088	3.3779
	Brown's agar	MCD	3.14000*	0.14694	0.000	2.7055	3.5745
	Dox agar	MCD	-4.87000*	0.14694	0.000	-5.3045	-4.4355
	Cerelose ammonium nitrate	MCD	-4.87000*	0.14694	0.000	-5.3045	-4.4355
	citrate agar medium	MCD	4.48667*	0.14694	0.000	4.0521	4.9212
	Kuehner's basal medium	MCD	-4.87000*	0.14694	0.000	-5.3045	-4.4355
	Piefer, Humphrey and Acree's medium	MCD	-4.87000*	0.14694	0.000	-5.3045	-4.4355
**C**	Scrose proline agar	MCD	-0.22667	0.12648	0.419	-0.6007	0.1473
	Mineral salts agar	MCD	-0.29667	0.12648	0.168	-0.6707	0.0773
	Asthana and Hawker medium A	MCD	0.43333*	0.12648	0.018	0.0593	0.8073
	Elliott's agar	MCD	-0.49333*	0.12648	0.006	-0.8673	-0.1193
	Brown's agar	MCD	1.26667*	0.12648	0.000	0.8927	1.6407

	Dox agar	MCD	0.05000	0.12648	1.000	-0.3240	0.4240
	Cerelose ammonium nitrate	MCD	-2.18333*	0.12648	0.000	-2.5573	-1.8093
	Citrate agar medium	MCD	0.80333*	0.12648	0.000	0.4293	1.1773
	Kuehner's basal medium	MCD	-2.24333*	0.12648	0.000	-2.6173	-1.8693
	Piefer, Humphrey and Acree's medium	MCD	-2.38667*	0.12648	0.000	-2.7607	-2.0127
**SAA10**	Scrose proline agar	MCD	-0.65667*	0.04420	0.000	-0.7874	-0.5260
	Mineral salts agar	MCD	-0.63333*	0.04420	0.000	-0.7640	-0.5026
	Asthana and Hawker medium A	MCD	-0.59000*	0.04420	0.000	-0.7207	-0.4593
	Elliott's agar	MCD	-0.62333*	0.04420	0.000	-0.7540	-0.4926
	Brown's agar	MCD	-0.65000*	0.04420	0.000	-0.7807	-0.5193
	Dox agar	MCD	-0.61333*	0.04420	0.000	-0.7440	-0.4826
	Cerelose ammonium nitrate	MCD	-2.22000*	0.04420	0.000	-2.3507	-2.0893
	Citrate agar medium	MCD	-0.68000*	0.04420	0.000	-0.8107	-0.5493
	Kuehner's basal medium	MCD	-2.22000*	0.04420	0.000	-2.3507	-2.0893
	Piefer, Humphrey and Acree's medium	MCD	-2.22000*	0.04420	0.000	-2.3507	-2.0893
**IIV3-3**	Scrose proline agar	MCD	0.35000	0.18474	0.360	-0.1963	0.8963
	Mineral salts agar	MCD	0.12667	0.18474	0.991	-0.4196	0.6730
	Asthana and Hawker medium A	MCD	0.66333*	0.18474	0.012	0.1170	1.2096
	Elliott's agar	MCD	0.44000	0.18474	0.157	-0.1063	0.9863
	Brown's agar	MCD	0.51333	0.18474	0.072	-0.0330	1.0596

	Dox agar	MCD	0.39000	0.18474	0.254	-0.1563	0.9363
	Cerelose ammonium nitrate	MCD	-0.84667*	0.18474	0.001	-1.3930	-0.3004
	Citrate agar medium	MCD	0.65333*	0.18474	0.014	0.1070	1.1996
	Kuehner's basal medium	MCD	-0.33667	0.18474	0.401	-0.8830	0.2096
	Piefer, Humphrey and Acree's medium	MCD	0.25000	0.18474	0.714	-0.2963	0.7963
**I27**	Scrose proline agar	MCD	-0.51000*	0.17157	0.048	-1.0174	-0.0026
	Mineral salts agar	MCD	1.42667*	0.17157	0.000	0.9193	1.9340
	Asthana and Hawker medium A	MCD	0.55333*	0.17157	0.028	0.0460	1.0607
	Elliott's agar	MCD	-0.97333*	0.17157	0.000	-1.4807	-0.4660
	Brown's agar	MCD	0.10667	0.17157	0.996	-0.4007	0.6140
	Dox agar	MCD	-0.35667	0.17157	0.268	-0.8640	0.1507
	Cerelose ammonium nitrate	MCD	-2.01000*	0.17157	0.000	-2.5174	-1.5026
	Citrate agar medium	MCD	0.60333*	0.17157	0.015	0.0960	1.1107
	Kuehner's basal medium	MCD	-2.01000*	0.17157	0.000	-2.5174	-1.5026
	Piefer, Humphrey and Acree's medium	MCD	-2.01000*	0.17157	0.000	-2.5174	-1.5026
**URA1**	Scrose proline agar	MCD	-0.04333	0.06833	0.995	-0.2454	0.1587
	Mineral salts agar	MCD	0.29333*	0.06833	0.002	0.0913	0.4954
	Asthana and Hawker medium A	MCD	0.01667	0.06833	1.000	-0.1854	0.2187
	Elliott's agar	MCD	0.10333	0.06833	0.603	-0.0987	0.3054
	Brown's agar	MCD	0.15000	0.06833	0.220	-0.0521	0.3521

	Dox agar	MCD	-0.89667*	0.06833	0.000	-1.0987	-0.6946
	Cerelose ammonium nitrate	MCD	-0.89667*	0.06833	0.000	-1.0987	-0.6946
	Citrate agar medium	MCD	3.01333*	0.06833	0.000	2.8113	3.2154
	Kuehner's basal medium	MCD	-0.89667*	0.06833	0.000	-1.0987	-0.6946
	Piefer, Humphrey and Acree's medium	MCD	-0.89667*	0.06833	0.000	-1.0987	-0.6946
**KhDS2-3**	Scrose proline agar	MCD	0.22667*	0.03898	0.000	0.1114	0.3419
	Mineral salts agar	MCD	0.25000*	0.03898	0.000	0.1347	0.3653
	Asthana and Hawker medium A	MCD	0.22667*	0.03898	0.000	0.1114	0.3419
	Elliott's agar	MCD	0.25000*	0.03898	0.000	0.1347	0.3653
	Brown's agar	MCD	0.25000*	0.03898	0.000	0.1347	0.3653
	Dox agar	MCD	-0.75000*	0.03898	0.000	-0.8653	-0.6347
	Cerelose ammonium nitrate	MCD	-0.75000*	0.03898	0.000	-0.8653	-0.6347
	Citrate agar medium	MCD	0.81000*	0.03898	0.000	0.6947	0.9253
	Kuehner's basal medium	MCD	-0.75000*	0.03898	0.000	-0.8653	-0.6347
	Piefer, Humphrey and Acree's medium	MCD	-0.75000*	0.03898	0.000	-0.8653	-0.6347
**EES2-2**	Scrose proline agar	MCD	2.70000*	0.16160	0.000	2.2221	3.1779
	Mineral salts agar	MCD	3.45333*	0.16160	0.000	2.9755	3.9312
	Asthana and Hawker medium A	MCD	0.00000	0.16160	1.000	-0.4779	0.4779
	Elliott's agar	MCD	3.40000*	0.16160	0.000	2.9221	3.8779
	Brown's agar	MCD	3.36333*	0.16160	0.000	2.8855	3.8412

	Dox agar	MCD	2.57000*	0.16160	0.000	2.0921	3.0479
	Cerelose ammonium nitrate	MCD	0.00000	0.16160	1.000	-0.4779	0.4779
	Citrate agar medium	MCD	3.74667*	0.16160	0.000	3.2688	4.2245
	Kuehner's basal medium	MCD	0.00000	0.16160	1.000	-0.4779	0.4779
	Piefer, Humphrey and Acree's medium	MCD	0.00000	0.16160	1.000	-0.4779	0.4779
**Zn8-2**	Scrose proline agar	MCD	0.00000	0.12470	1.000	-0.3688	0.3688
	Mineral salts agar	MCD	7.48000*	0.12470	0.000	7.1112	7.8488
	Asthana and Hawker medium A	MCD	0.00000	0.12470	1.000	-0.3688	0.3688
	Elliott's agar	MCD	0.00000	0.12470	1.000	-0.3688	0.3688
	Brown's agar	MCD	5.72333*	0.12470	0.000	5.3546	6.0921
	Dox agar	MCD	0.00000	0.12470	1.000	-0.3688	0.3688
	Cerelose ammonium nitrate	MCD	0.00000	0.12470	1.000	-0.3688	0.3688
	Citrate agar medium	MCD	7.41667*	0.12470	0.000	7.0479	7.7854
	Kuehner's basal medium	MCD	0.00000	0.12470	1.000	-0.3688	0.3688
	Piefer, Humphrey and Acree's medium	MCD	0.00000	0.12470	1.000	-0.3688	0.3688
**G88**	Scrose proline agar	MCD	0.00000	0.04878	1.000	-0.1443	0.1443
	Mineral salts agar	MCD	0.94667*	0.04878	0.000	0.8024	1.0909
	Asthana and Hawker medium A	MCD	0.00000	0.04878	1.000	-0.1443	0.1443
	Elliott's agar	MCD	0.00000	0.04878	1.000	-0.1443	0.1443
	Brown's agar	MCD	0.00000	0.04878	1.000	-0.1443	0.1443

Dox agar	MCD	0.00000	0.04878	1.000	-0.1443	0.1443
Cerelose ammonium nitrate	MCD	0.00000	0.04878	1.000	-0.1443	0.1443
Citrate agar medium	MCD	2.66667*	0.04878	0.000	2.5224	2.8109
Kuehner's basal medium	MCD	0.00000	0.04878	1.000	-0.1443	0.1443
Piefer, Humphrey and Acree's medium	MCD	0.00000	0.04878	1.000	-0.1443	0.1443

"*" indicates that the mean difference is significant at the 0.05 level. "-" in mean difference section indicates to less enzyme production in certain culture medium in comparison to basal culture medium (MCD culture medium).
