# Supplementary figures and images for: L‒asparaginase activity in some endophytic fungi: Glutaminase‒free and low urease co‒activities
Source: PLoS One. 2026 Feb 13;21(2):e0339829. doi: 10.1371/journal.pone.0339829 (PMC12904411; doi:10.1371/journal.pone.0339829)

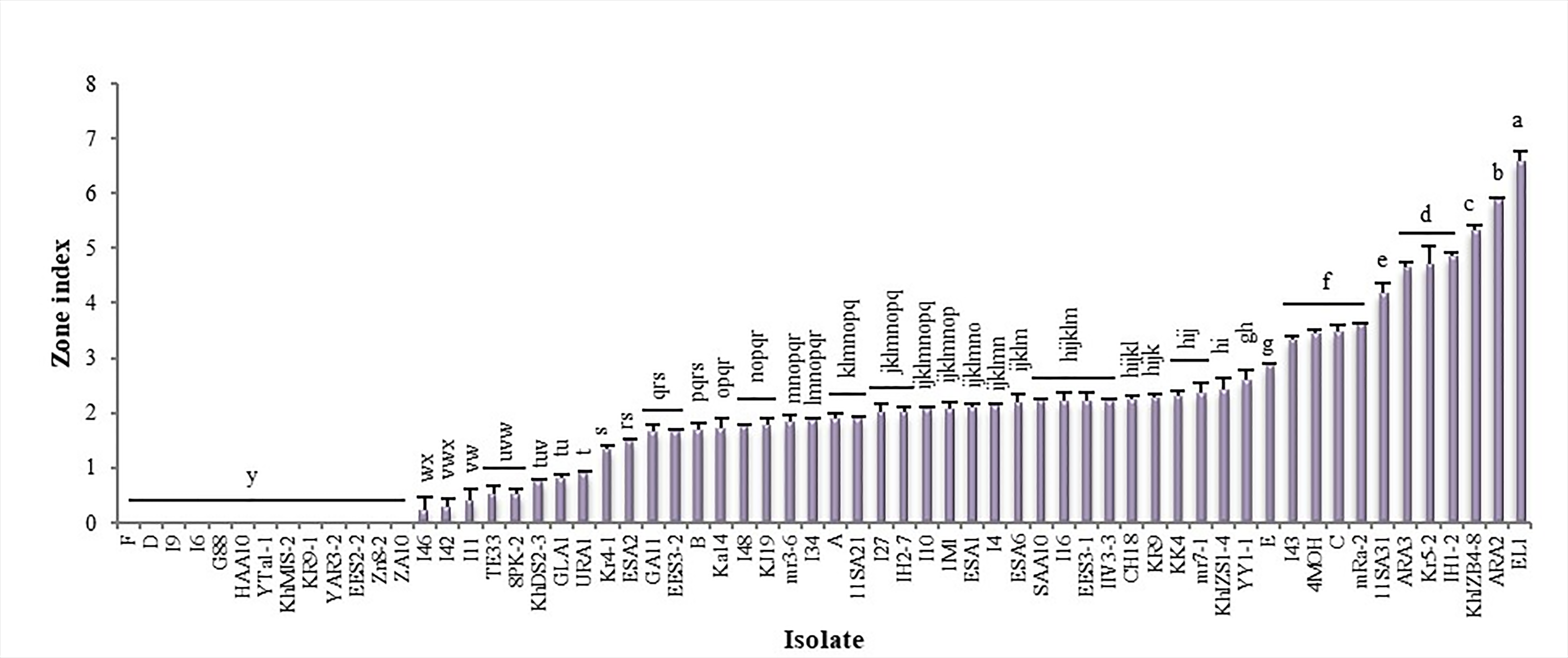

Supplement: S1 Fig — Mean zone index for each isolate is presented. Isolates were grouped based on Duncan’s post hoc test at a significance level of 0.05 (p‒value = 3.5 × 10^-87^). Letters above each column indicate the groups identified by Duncan’s analysis; isolates sharing the same letter belong to the same group. Error bars represent the standard error. (TIF) [file pone.0339829.s006.tif]

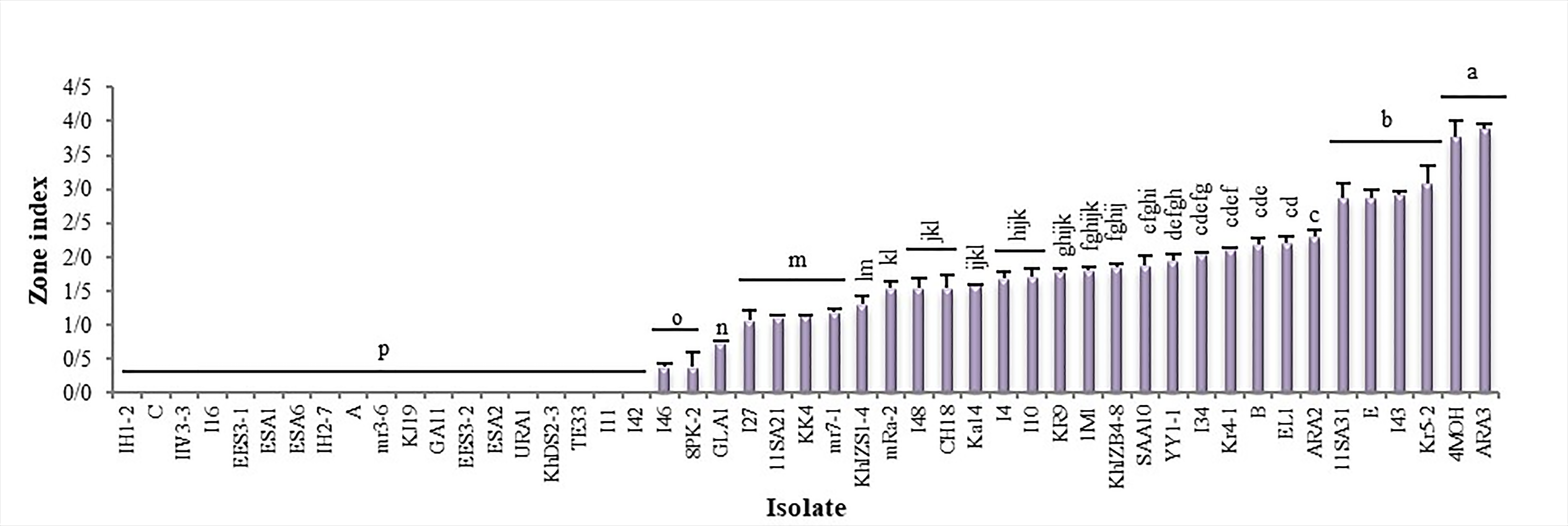

Supplement: S2 Fig — Mean zone index for each isolate is presented. Isolates were grouped using Duncan’s post hoc test at a significance level of 0.05 (p-value = 1.4 × 10^-49^). Letters above each column indicate groupings based on Duncan’s analysis; isolates sharing the same letter belong to the same group. Error bars represent the standard error. (TIF) [file pone.0339829.s007.tif]

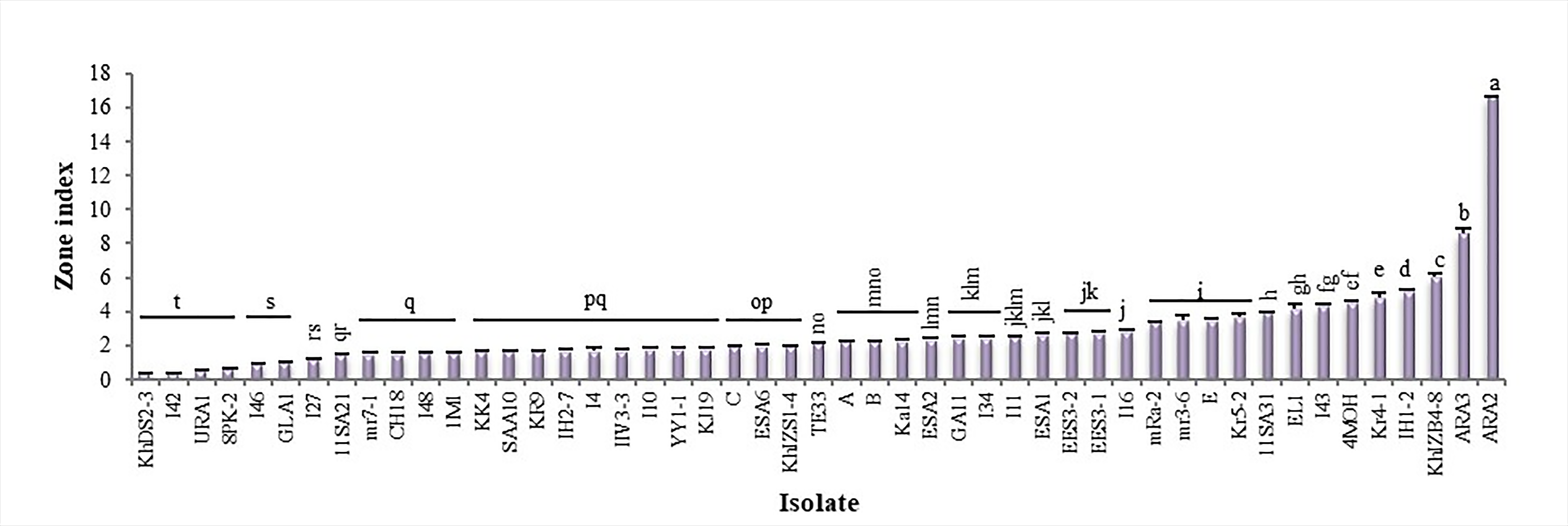

Supplement: S3 Fig — Mean zone index for each isolate is presented. Isolates were grouped using Duncan’s post hoc test at a significance level of 0.05 (p-value = 1.0 × 10-108). Letters above each column indicate groupings based on Duncan’s analysis; isolates sharing the same letter belong to the same group. Error bars represent the standard error. (TIF) [file pone.0339829.s008.tif]
